# Erythrocyte membrane in type 2 diabetes mellitus

**DOI:** 10.15190/d.2016.7

**Published:** 2016-06-30

**Authors:** Georgiana Roxana Gabreanu, Silvana Angelescu

**Affiliations:** Hematology Department, Coltea Clinical Hospital, Bucharest, Romania

**Keywords:** type 2 diabetes mellitus (T2DM), erythrocyte membrane, non-enzymatic glycation of proteins, Amadori compounds, advanced glycated end products (AGEs), lipid peroxidation, lipid rafts, erythrocyte membrane fatty acids, erythrocyte membrane pumps

## Abstract

Type 2 diabetes mellitus represents a major public health challenge, due to the continuously growing prevalence and the complexity of the diabetic complications. Hyperglycemia seems to be the main mechanism for the disease progression. During erythrocyte’s long life span, erythrocyte membranes are affected by the chronic exposure to glucose, which triggers several biochemical modifications that lead to both structural and functional disruption, which are further involved in the physiopathology of diabetes and its complications. Non-enzymatic protein glycation of red blood cell membrane proteins occur in two phases: early glycation, characterized by Schiff bases and Amadouri compounds formation, and advanced glycation, characterized by advanced glycation end products (AGEs). These products could be valuable tools for early diagnosis or biomarkers for disease progression, depending on how advanced they are in the glycation process. Advanced glycated end products were linked with diabetic complications. Also, lipid peroxidation and decreased activity of the enzyme pumps occur in the erythrocyte membrane of the diabetic patients. The investigation of lipid rafts and erythrocyte membrane fatty acids are a valuable tool for long-term monitoring of metabolic status. Further investigation of the erythrocyte membrane could provide novel biomarkers for monitoring of diabetes and its complications.

## 1. Introduction

Type 2 diabetes mellitus represents a major public health challenge, due to the continuously growing prevalence. According to the International Federation of Diabetes, there was an increase by around 64% in the worldwide prevalence, from 2000 to 2015 (from estimated 151 million to 415 million, respectively), and it is expected to surge from this point by 36% until 2040, reaching about 642 million^1,2^. The most common form of diabetes mellitus is represented by type 2 (up to 91 % in high-income countries)^2^. The pathogenesis of diabetic complications is complex, but hyperglycemia seems to be the main mechanism for the disease progression. Chronic exposure to glucose affects all the human cells. However, the erythrocytes gained special interest for the research community due to their long life span of 120 days^3^.

## 2. Non-enzymatic protein glycation

Non-enzymatic glycation of proteins (also known as browning reaction) was initially described by the French chemist L.C. Maillard in 1912, being the first to question the implications of chemical reaction between amino acids and sugar in human physiology and pathology^4,5^. After two decades, the Maillard reaction began to be extensively researched, being highly involved in the development of the food industry^5^.

The first evidence of *in vivo* non-enzymatic protein glycosylation was brought in the 1960s, with the discovery of glycated hemoglobin (HbA1c) within the erythrocytes, which was a tremendous step in the understanding of diabetes pathophysiology and its complications, since HbA1c is currently the gold standard of diabetes management^6-9^. Nowadays, glycated hemoglobin is widely used as a biomarker for long-term glycemic control in patients with diabetes mellitus, indicating the average blood glucose level over a period of approximately 90 days, being able to detect chronic rather than acute exposure to high glucose concentrations. It was also reported as a sensitive risk biomarker for the microvascular complications of diabetes mellitus. Despite the obvious advantages, glycated hemoglobin has its limitations since several factors independent on glycemic status have been reported to influence the glycated hemoglobin levels, such as genetic influence, patient ethnicity, hematological diseases with rapid turnover of the red blood cells (anemia, hemolysis, hemoglobin pathologies and transfusions), individual variability of erythrocyte life span, age, pregnancy, vitamin C and E consumption and other medical conditions (malignancies, chronic liver disease, hemodialysis, uremia)^10,11^.

Due to a shorter life span of around 15-21 days, glycated serum albumin detects the glycemic fluctuations more rapidly than glycated hemoglobin, being useful as a short-term marker of glycemic control. While HbA1c brings an overall insight into the average glucose concentration during the past three months, glycated serum albumin is a more sensitive biomarker for early detection of glucose level changes, thus being more useful in the treatment management of patients with diabetes mellitus^12,13^.Otherglycated plasma proteins were also found to be upregulated in patients with diabetes and impaired glucose tolerance versus control: abundant plasma proteins, such as serotransferrin, alpha-1-antitrypsin, alpha-2-macroglobulin, apolipoprotein A-I and A-II, fibrinogen, and alpha-1-acid glycoprotein and moderately abundant plasma proteins, such as ceruloplasmin, complement C3 and C4A precursors, kininogen-1 precursor, vitamin D-binding protein precursor, apolipoprotein B-100 and lumican precursors^14^.

Both glycated hemoglobin and glycated serum albumin are produced from the condensation of glucose with hemoglobin, respectively serum albumin, with the formation of a reversible aldimine (namely Schiff base), which is a precursor of the glycated products. Normally, the process of non-enzymatic glycation begins with the condensation of a protein through its amino-terminal group with the carbonyl group of a reducing sugar. Amadori rearrangement occurs in the Schiff base resulted, which further leads to the formation of Amadori products. The proportion of glycation is directly proportional to time-averaged glucose concentrations and Amadori products accumulate in proportion to average glucose concentrations.

Further oxidation occurs to form the advanced glycated end products (AGE). Another pathway for AGE formation is the non-oxidative degradation to alfa-dicarbonyl intermediates, with subsequent production of carboxylmethyllysine or hydroimidazolone. AGEs are irreversible late glycation products known to be involved in the micro and macro-vascular complications of diabetes mellitus^12,15,16^. Structural and functional modifications are triggered in the glycated proteins. For example, the transport function and the antioxidant properties of the glycated human serum albumin are disrupted^12^.

## 3. Glycated erythrocyte membrane proteins: potential biomarkers in type 2 diabetes

**Figure 1:**
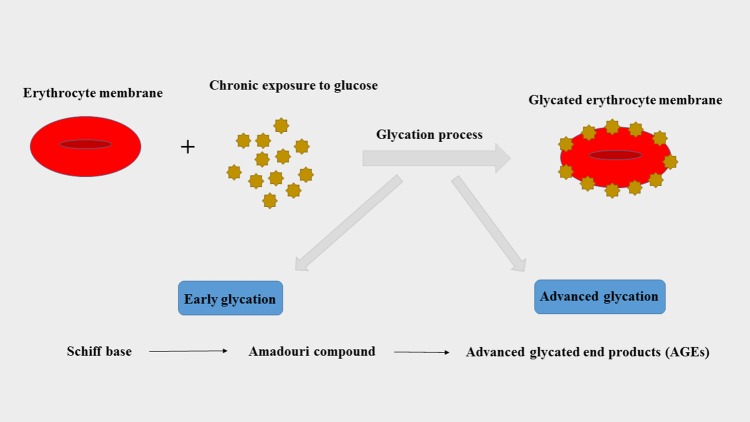
The glycation process of erythrocyte membrane Chronic exposure to glucose triggers the non-enzymatic glycation of the erythrocyte membrane proteins, with the formation of early glycation products, such as Schiff base and Amadouri compound and advanced glycation products, such as AGEs.

It is well known that the red blood cells have longer life span compared to other cells, making them more susceptible to accumulation of Amadouri compound when exposed to high glucose concentrations during their life cycle. However, glycated hemoglobin reflects the level of non-enzymatic glycation inside the red blood cell, thus is more predisposed to variations in case of disrupted cellular glucose transport. It has been suggested that the erythrocyte membrane proteins are in closer contact with glucose, making the process of glycation much easier. The glycation process of erythrocyte membrane proteins in chronic hyperglycemic conditions is schematically represented in**Figure 1**. Thus, the red blood cell membrane could provide more sensitive biomarkers of glycemic control after non-enzymatic glycation of its proteins^14^.

Erythrocyte membrane proteins are composed of integral proteins, which are important elements with structural functions, such as band 3 and glycophorins. Integral proteins are strongly embedded into the lipid layer, making them harder to be extracted from the membrane, thus only harsh reagents, such as detergents can detach them for separate investigation. The peripheral proteins, are much easier to isolate with salts (high or low) and pH extraction (high) and can be divided into cytoskeletal proteins (spectrin, actin, protein 4.1) and anchoring proteins (ankyrin, protein 4.2). Anchoring proteins are forming the “band 3 macro complex” and function like a bridge by interacting with the integral protein and cytoskeletal proteins. Protein kinase A, casein kinase I and cyclic AMP-independent protein kinase modulate these interactions by phosphorylation of ankyrin. Similarly, the cytoskeletal proteins are known to form the “junctional complex”, with 4.1R component having a key role by interaction with both integral proteins (band 3 and glycophorins C/D) and cytoskeletal proteins (spectrin and actin) It is well known that protein kinase C and A, calmodulin, casein kinase can decrease these interactions by phosphorylation of 4.1R protein, triggering the red blood cell membrane deformability^17^.

Modern proteomics approach made possible the first description of the glycated proteome in the erythrocyte membranes of patients with type 2 diabetes mellitus and impaired glucose tolerance versus individuals with normal glucose tolerance. Glycated proteins and peptides were enriched using phenylboronate affinity chromatography, and then analyzed by liquid chromatography coupled with electron transfer dissociation-tandem mass spectrometry^14^.Updates were reported by the same research team several years after, attempting to create a database as a tool for novel biomarkers research in diabetes^18^.

However, authors didn’t found significant differences between diabetes and impaired glucose tolerance groups, but taken together as a whole group described as abnormal glucose tolerance, differences aroused compared with controls. Several glycated erythrocyte membrane proteins were found to be upregulated in individuals with abnormal glucose tolerance compared to control: actin (alpha skeletal muscle and aortic smooth muscle), protein 4.1 (isoform 1), spectrin alpha, spectrin beta chain (isoform 2), protein band 4.2 (isoform long) and ankyrin 1. Erythrocyte cytoplasm was also investigated and upregulated glycated glyceraldehyde-3-phosphate dehydrogenase (GAPDH), carbonic anhydrases (1 and 2) and hemoglobin (subunits alpha, beta and delta) were described.

The results are not surprising since hyperglycemia favors the process of non-enzymatically glycation, but clinicians should interpret the list of newly discovered glycated proteins with caution, as the authors indicated, due to individual hereditary variations in glycated hemoglobin levels and blood glucose metabolism that may be a source of bias^14,18^.

## 4. Advanced glycated end products (AGEs)

Several advanced glycation end products were identified in the investigation of various samples from diabetic patients, such as imidazolone, crossline, pyrraline, N epsilon-(Carboxymethyl)lysine (CML) and pentosidine, which were linked with the development of diabetic complications^19-23^. AGEs proved to be useful as biomarkers for type 2 diabetic patients. For example, serum pentosidine measured by ELISA assay was found elevated in both diabetic nephropathy and retinopathy, and it was suggested as a useful biomarker for microvascular complications in type 2 diabetes^24^.

Advanced glycated end products (AGEs) were also identified in the erythrocyte peripheral membrane proteins of 48 diabetic subjects, using an ELISA assay with specific antibodies against AGE. It seems that the late glycation products were increased by 3-fold in the diabetic patients group compared with healthy controls, and the results correlated with the glycated hemoglobin levels. The investigation through SDS-PAGE analysis and immunoblotting identified spectrin as a major component of the erythrocyte peripheral membrane proteins and suggested the implication of AGEs in the disparities that occur in the diabetic red blood cells, such as decreased deformability and reduced membrane fluidity^25^. It has been observed that diabetic patients present high levels of AGEs on the surface of their red blood cells, which are capable to interact with an immunoglobulin expressed on endothelial cells, named receptor for AGE (RAGE), thus triggering a vascular dysfunction that is involved in the diabetic vascular complications^26^. Crossline is a fluorescent advanced glycated end product. Crossline levels from erythrocyte membrane proteins were measured by an enzyme-linked immunosorbent assay (ELISA), with an increase by 1.6-fold reported in diabetic patients compared with healthy controls and significantly higher levels by 2.2-fold (non-proliferative retinopathy) and 2.6-fold (proliferative retinopathy) described in the patients with complicated diabetes. Authors suggest that crossline levels of erythrocyte membrane proteins could provide a useful marker for the progression of diabetic retinopathy^20^.In addition, crossline levels from erythrocyte membrane was also linked with diabetic nephropathy, since enhanced concentrations were found in patients with diabetic nephropathy compared with those without clinical proteinuria. However, the authors suggest the investigation of serum crossline in parallel, for a better staging of the diabetic nephropathy, since serum crossline levels were higher in the advanced versus the moderate stages of the disease^27^ (**Table 1**).

## 5. Lipid peroxidation in the erythrocyte membrane

Erythrocyte membrane lipids are composed of 60% of phospholipids, 30% non-esterified cholesterol and 10% glycolipids. The majority of phospholipids are phosphatidylcholine (PC), phosphatidylethanolamine (PE), sphingomyelin (SM) and phosphatidylserine (PS) and the minor component is represented by phosphatidylinositol (PI), PI-monophosphate (PIP), PI-4,5-bisphosphate (PIP2), phosphatidic acid (PA), lysophosphatidylcholine (Lyso-PC) and lysophosphatidylethanolamine (Lyso-PE). As any other membrane, the red blood cell membrane is normally characterized by the asymmetrical distribution of lipids, feature that has an important role in both structure and function of the erythrocyte. For example, glycolipids (such as glycosphingolipids) are mainly distributed on the exterior surface of the membrane, and are responsible for the adhesiveness of the red blood cell to the extracellular environment, due to the sugar residues. Also, phosphatidylcholine and sphingomyelin are normally exposed on the outer surface, while phosphatidylserine and phosphatidylethanolamine are found on the inner leaflet^17^.

Chronic hyperglycemia in diabetes disrupts the asymmetry of the membrane and shortens the life span of the erythrocyte by triggering the exposure of phosphatidylserine to the outer surface of the membrane and thus, making it recognizable for the engulfing macrophages. One of the pathways seems to be the accumulation of the glucose metabolite methylglyoxal^28^. Also, enhanced Ca^2+ ^intracellularlevels triggers the erythrocyte death, which has been named eryptosis, due to the lack of mitochondria or nucleus in the red blood cells, in order to differentiate from apoptosis of the nucleated cells^29^. Enhanced oxidative stress on lipids was found in a group of diabetic patients, with the highest levels in the diabetic subjects complicated with nephropathy. In contrast, the group of diabetic patients with nephropathy showed lower concentrations of antioxidants, such as erythrocyte GSH, and lower erythrocyte GSH/GSSG ratio, compared to healthy controls or patients with uncomplicated diabetes. Interestingly, the phosphatidylserine externalization, as a marker for eryptosis, was positively correlated with the level of lipid peroxidation, while negatively correlated with both erythrocytes GSH/GSSG ratio and total plasma antioxidant capacity^30^(**Table 1**).

Chronic hyperglycemia triggers glycation in several compounds of the erythrocyte membrane, and further oxidation on the glycated products generate the development of the oxygen-derived free radicals, which could be responsible for the damaging oxidative stress, involved in the diabetes pathology. The investigation of erythrocyte membrane through gas chromatography–mass spectrometry provided several biomarkers of lipid peroxidation in diabetic patients^31-33^ (**Table 1**). For example, significantly increased ratios of conjugated linoleic acid to linoleic acid were described in patients with diabetes, compared with control and the results correlated with glycated hemoglobin, which indicated a possible link of glycation due to chronic hyperglycemia with lipid peroxidation^31^.The correlation of glycated hemoglobin levels with the increased ratio of one of the major cholesterol peroxidation product, 7-oxocholesterol, to cholesterol concentrations is another evidence of link between glycation and lipid peroxidation^32^.Minor cholesterol peroxidation compounds were also useful as a marker for lipid peroxidation in diabetes patients, since significantly increased ratios of 3-cholesten-6-one to cholesterol were reported and correlation of these results with glycated hemoglobin levels was also described^33^.

## 6. Erythrocyte lipid rafts

Erythrocyte lipid rafts are micro-domains with lower fluidity compared with other membranes, as they are enriched in cholesterol and glycosphingolipids, such as gangliosides and sulfatides, with saturated fatty acyl chains. Lipid rafts have low density and are mainly composed of lipids, being therefore insoluble in cold nonionic detergents. For this reason, they are also known as detergent-resistant membranes (DRM), triton-insoluble membranes (TIM) or triton-insoluble floating fractions (TIFF). Only a small percent of the total red blood cell membrane proteins (4%) is embedded in the lipid-rich micro-domains, with three major integral proteins involved, such as: stomatin, flotillin-1 and flotillin-2. Other proteins were also described in the lipid rafts, such as duffy protein receptor and glycosylated phosphatidylinositol (GPI) - anchored proteins (CD55, CD58, CD59). Lipid rafts with specific signaling roles have been described, called caveolae, because of the augmented levels of caveolin protein^17^.

One of the major integral protein of erythrocyte lipid raft, flotillin-1 was reported to be affected in type 2 diabetes, after human red blood cells membranes were investigated using a proteomic approach by two-dimensional electrophoresis. In addition, abnormal syntaxin 1C and arginase were also described in patients with diabetes^34^. It seems that arginase and flotillin-1 are both elevated in erythrocyte membranes of type 2 diabetic patients, and flotillin-1 mediates the arginase binding to the membrane, also enhancing its activity^35^ (**Table 1**).

## 7. Erythrocyte membrane fatty acids (EMFA) in type 2 diabetes

The majority of erythrocyte fatty acids are normally represented by palmitic acid (16:0), stearic acid (18:0), which are saturated fatty acids, and arachidonic acid (20:4), oleic acid/elaidic acid/ vaccenic acid (18:1) and linoleic acid/linoelaidic acid (18:2), which are unsaturated fatty acids.The nomenclature of fatty acids suggest the number of carbons (represented by the first number: 16, 18, 20) and the number of double bounds (represented by the second number, which is 0 for saturated fatty acids and 1 or more for unsaturated fatty acids)^17^.

Omega 6 and omega 3 polyunsaturated fatty acid (PUFA) are precursors of important lipid regulators of inflammation (since “n-“ prefix is commonly used instead of “omega”, we will refer them as n-6 PUFA and n-3 PUFA, respectively). Eicosanoids resulting from n-6 PUFAs, such as arachidonic acid (AA), are known to induce a pro-inflammatory status, while eicosanoids resulting from n-3 PUFAs, such as eicosapentaenoic acid (EPA) and docosahexaenoic acid (DHA), have opposite anti-inflammatory effects^36^. The investigation of plasma PUFA profile by capillary gas chromatography, in a study performed on 396 patients with type 2 diabetes and 122 healthy controls, revealed decreased levels of EPA in diabetic patients versus controls, while elevated levels of DHA, AA, and dihomo-γ-linolenic acid (DGLA) were found. Therefore, the balance between n-3 and n-6 PUFAs was affected in the patients with diabetes, marked by decreased ratios EPA/AA, DHA/AA, and (EPA + DHA)/AA^37^. The results might indicate an inflammatory status in patients with diabetes, which would not be surprising, since there is an increasing evidence for the involvement of chronic inflammation in the pathology of diabetes and its complications^38-41^.

However, since fatty acids in plasma could be influenced by dietary intake, the measurement of erythrocyte membrane PUFA levels may be more useful compared to plasma lipids due to the erythrocyte’s stability during its long lifespan of 120 days, which makes possible the constant distribution of the fatty acids throughout the erythrocyte’s life cycle^42^. Moreover, the necessary enzymes for fatty acids metabolism are lacking in the red blood cell membrane, thus the erythrocyte membrane fatty acids are considered by several authors as unbiased biomarkers for the dietary fat intake, comparing with dietary questionnaires^43^. Taken all these advantages in consideration, it is suggested that red blood cell membranes could provide better indicators of long-term fatty acids profile compared to plasma lipids^42,43^.

Fatty acids from both plasma and erythrocyte membrane were investigated using gas chromatography-flame ionization detector (GC-FID) in patient groups versus healthy controls. High levels of long chain saturated fatty acids (palmitic acid and stearic acid) were found in both plasma and erythrocyte membrane in type 2 diabetes (with and without end stage renal disease), compared to end stage renal disease alone and healthy control groups, while decreased plasma levels of very long chain saturated fatty acids (behenic and lignoceric acids) were found in type 2 diabetes. Furthermore, both plasma and red blood cell membrane arachidonic acid were significantly higher in the diabetes group compared with controls. Positive correlations between plasma levels of palmitic acid, oleic acid and arachidonic acid in contrast with negative correlations between erythrocyte membrane levels of EPA and glycated hemoglobin levels were described. Thus, it has been indicated that red blood cell should be investigated in parallel with plasma fatty acids, due to the aforementioned advantages, but also because it is an accessible tool for research which could serve as a model for cell membranes research in diabetes^44^. Moreover, a prospective 5-year follow-up study on 1346 Finnish men described significant modifications on erythrocyte membrane fatty acids as good biomarkers for the risk evaluation of type 2 diabetes. Several indicators were associated with the worsening of hyperglycemia: palmitoleic acid, dihomo-γ-linolenic acid, ratio of palmitoleic acid (16:1n–7) to palmitic acid (16:0) as a marker of stearoyl coenzyme A desaturase 1 activity and the ratio of dihomo-γ-linolenic acid (20:3n−6) to linoleic acid (18:2n−6) as a marker of delta 6 desaturase activity. In contrast, decreased hyperglycemia was associated with linoleic acid and ratio of vaccenic acid (18:1n–7) to palmitoleic acid (16:1n–7) as a marker of elongase activity^45^ (**Table 1**).

## 8. Erythrocyte membrane pumps: impaired functions in type 2 diabetes

### Erythrocyte membrane Na+/K+-ATPase enzyme

The erythrocyte membrane Na+/K+-ATPase enzyme activity is significantly reduced in both insulin-dependent and non-insulin-dependent diabetic human patients. At first, the mechanisms seemed to be other than hyperglycemia, since no correlation was found between Na+/K+-ATPase activity and glycated hemoglobin or blood glucose concentrations^46^. However, a recent study performed on three groups of individuals (healthy controls and diabetes mellitus type 2, with or without metabolic syndrome) reported contrasting evidence of negative correlation between HbA1c and the erythrocytes enzyme pump. The authors claim that the differences might come from the correlation analysis, and they suggest that all the subjects investigated in the study should be taken into consideration for the analysis^47^.

Erythrocyte membrane’s viscosity and fluidity, among other factors, such as lipid peroxidation, protein glycation or oxidative stress effects on proteins were suggested to be possible causes of decreased activity in the erythrocyte pump. Consequently, the permeability of the membrane could be affected, which might lead to abnormal electrolytes levels^47,48^. For example, a study performed on 60 patients with diabetes showed reduced activity of erythrocyte membrane Na+/K+ pump, with low levels of potassium and accumulation of sodium inside the erythrocyte cell, compared to controls. Furthermore, serum electrolytes were measured by flame photometer and spectrophotometer, and decreased concentrations of magnesium, while enhanced levels of both sodium and potassium were reported in diabetic patients versus controls^49^.

The red blood cell membrane lipids are also significantly affected, since increased cholesterol: phospholipid ratio was observed in diabetic patients compared with healthy controls, regardless of dyslipidemia association. Abnormal ratio between cholesterol and phospholipids disrupts the fluidity of membrane, affecting the erythrocyte’s morphology which leads to diminished life span of red blood cells in the diabetic patients^47^.Thus, it is not surprising that a significant correlation between enzyme’s activity and erythrocyte membrane fluidity was found^46^.

It seems that a poor metabolic control is also linked with the reduced activity of Na+/K+ pump in diabetic patients, since the erythrocyte membrane pump was positively correlated with the high density lipoprotein cholesterol (HDL-C) and negatively correlated with triacylglycerol (TGs). In contrast, no significant association was found between the enzyme activity and high sensitivity C-reactive proteins, body mass index, systolic and diastolic blood pressure^47^(**Table 1**).

### Erythrocyte sodium-lithium countertransport (SLC)

Erythrocyte sodium-lithium countertransport (SLC) has been indicated as a good predictor of hypertension, therefore a useful biomarker for early detection of nephropathy in type 2 diabetes^50^. Insights into the possible pathways underlying could be provided by the significant correlation of increased sodium-lithium countertransport activity with upregulated intracellular sodium concentration and intracellular free calcium-ion concentration described in a study performed on 48 patients with type 2 diabetes and 24 healthy controls^51^. It seems that thiol protein alkylation with N-ethtyl maleimide (NEM) could be one of the mechanisms involved in the impaired regulation of erythrocyte sodium-lithium countertransport and abnormal membrane fluidity in some patients with type 2 diabetes^52,53^(**Table 1**).

**Table 1:** Markers of erythrocyte membrane modifications in type 2 diabetes

Marker	Observation	Reference
Crossline	Advanced glycation end product (AGE) Increased in diabetic patients, with higher concentrations in complicated diabetes (retinopathy, nephropathy)	^20,27^
Phosphatidylserine exposure	Marker of eryptosis (erythrocyte death) Triggered by chronic hyperglycemia due to the accumulation of methylglyoxal or by enhanced Ca2+ intracellular levels Positively correlated with the level of lipid peroxidation Negatively correlated with erythrocytes GSH/GSSG ratio and total plasma antioxidant capacity	^28-30^
Conjugated linoleic acid to linoleic acid ratio	Increased ratio in diabetic patients Marker of lipid peroxidation	^31^
7-Oxocholesterol to cholesterol ratio	Increased ratio in diabetic patients Marker of lipid peroxidation	^32^
3-cholesten-6-one to cholesterol ratio	Increased ratio in diabetic patients Marker of lipid peroxidation	^33^
Flotillin-1	One of the major integral protein of erythrocyte lipid raft Elevated levels in erythrocyte membranes of T2DM patients Mediates the arginase binding to the membrane, also enhancing its activity	^34,35^
Long chain saturated fatty acids (palmitic acid and stearic acid)	Erythrocyte membrane fatty acids High levels in plasma and erythrocyte membrane in T2DM	^44^
Arachidonic acid	Erythrocyte membrane fatty acid Eicosanoid resulting from n-6 PUFAs, known to induce a pro-inflammatory status High levels in both plasma and red blood cell membrane in T2DM	^36,44^
Eicosapentaenoic acid (EPA)	Erythrocyte membrane fatty acid Eicosanoid resulting from n-3 PUFAs, known to induce an anti-inflammatory effect Negative correlations between erythrocyte membrane levels of EPA and glycated hemoglobin in T2DM	^36,44^
Palmitoleic acid	Erythrocyte membrane fatty acid Associated with the worsening of hyperglycemia	^45^
Dihomo-γ-linolenic acid	Erythrocyte membrane fatty acid Associated with the worsening of hyperglycemia	^45^
Palmitoleic acid (16:1n–7) to palmitic acid (16:0) ratio	Erythrocyte membrane fatty acids Associated with the worsening of hyperglycemia Marker of stearoyl coenzyme A desaturase 1 activity	^45^
Dihomo-γ-linolenic acid (20:3n−6) to linoleic acid (18:2n−6)	Erythrocyte membrane fatty acids Associated with the worsening of hyperglycemia Marker of delta 6 desaturase activity	^45^
Linoleic acid	Erythrocyte membrane fatty acid Associated with decreased hyperglycemia	^45^
Vaccenic acid (18:1n–7) to palmitoleic acid (16:1n–7) ratio	Erythrocyte membrane fatty acids Associated with decreased hyperglycemia Marker of elongase activity	^45^
Na+/K+-ATPase enzyme	Erythrocyte membrane pump Significantly reduced in diabetic patients, due to lipid peroxidation, protein glycation or oxidative stress effects Abnormal electrolytes levels: low levels of potassium and accumulation of sodium inside the erythrocyte cell Linked with a poor metabolic control: positively correlated with the high density lipoprotein cholesterol (HDL-C) and negatively correlated with triacylglycerol (TGs)	^46-49^
Erythrocyte sodium-lithium countertransport (SLC)	Erythrocyte membrane pump Increased sodium-lithium countertransport activity Thiol protein alkylation with N-ethtyl maleimide (NEM) is one of the mechanisms for SLC impairment in T2DM A good predictor of hypertension A useful biomarker for early detection of nephropathy in type 2 diabetes	^50-53^

### Ca2+ ATPase

Non-enzymatic glycation of Ca^2+^ pump within erythrocyte membrane was suggested to be a mechanism for the inactivation of Ca^2+^ ATPase as a result of exposure to high glucose concentration, but further studies are needed for a more detailed description of glycation steps^54^. Once glycated at a single essential lysine residue, probably located near the catalytic site of the Ca^2+^ ATPase enzyme, the integral membrane protein is completely inactivated, losing its capacity to hydrolyze ATP, to be phosphorylated and to pump Ca^2+^ out of the cell cytoplasm, with possible subsequent accumulation of Ca^2+^ within the erythrocyte^55^. However, contrasting results were reported when Ca^2+^ extrusion rate showed no significant difference between diabetic and non-diabetic subjects, and no inhibition was observed in the Ca^2+^ pump when intact human and rat erythrocytes were exposed to high glucose in both in vivo and in vitro^56^. The activity of Ca2+ pump was reported to be dependent on the erythrocyte’s age, but the mechanisms by which the enzyme’s activity is decreased are not linked to glycation, since there were no differences regarding the enzyme’s activity between young and old erythrocytes after exposure to high glucose^57,58^. Overall, the authors conclude that in intact red blood cells the ATP could protect the pump from in vivo glycation, and such a protection could be lost in isolated membrane preparations, due to the disruption of the cell^55-58^.However, it has been shown that Ca^2+^ homeostasis is altered in patients with type 2 diabetes mellitus, thus further investigations are required for the Ca^2+ ^ATPase pump, which is a critical regulator of Ca^2+^ concentrations within cells, including erythrocytes^59^.

## 9. Conclusions

In diabetes, erythrocyte membranes are affected by the chronic exposure to glucose, and several biochemical modifications are triggered, with subsequent structural and functional disruption of the red blood cell, which are further involved in the physiopathology of diabetes and its complications. Due to the long lifespan of the erythrocytes (120 days), the red blood cell membrane could provide more sensitive markers for the long-term glycemic control. The main non-enzymatic protein glycation products might be useful biomarkers for early diagnosis or disease progression, depending on how advanced they are in the glycation process. Late glycation products, such as advanced glycated end products were linked with diabetic complications, and might be useful for clinicians in monitoring the progression of the disease. Furthermore, a better understanding of early glycation products in the erythrocyte membrane, such as Schiff bases and Amadouri compounds, could be suitable for early diagnosis. The investigation of lipid peroxidation, lipid rafts and erythrocyte membrane fatty acids are also a promising tool for long-term monitoring of metabolic status. Further research on the erythrocyte membrane could provide new biomarkers for monitoring of diabetes and its complications.

## Bullet Points

**◊** **Investigation of the erythrocyte membrane brings new insights in the physiopathology of type 2 diabetes mellitus and its complications.**

**◊** **Further research on the non-enzymatic glycation process in the red blood cell membrane could provide new biomarkers for the long-term glycemic control in diabetes.**

**◊** **The investigation of lipid peroxidation, lipid rafts and erythrocyte membrane fatty acids could be a promising tool for long-term monitoring of metabolic status in diabetes.**

## Open Questions

**◊** **Has the erythrocyte membrane the potential to be included in the routine laboratory investigations for type 2 diabetes mellitus?**

**◊ Is it clinical relevant and cost-effective to consider the investigation of the red blood cell membrane in the diabetes management?**
